# Extreme levels of mycophilia documented in Mazovia, a region of Poland

**DOI:** 10.1186/s13002-019-0291-6

**Published:** 2019-02-12

**Authors:** Marcin Andrzej Kotowski, Marcin Pietras, Łukasz Łuczaj

**Affiliations:** 1 Department of Botany, Faculty of Biotechnology, University of Rzeszów, Pigonia 1, 35-310 Rzeszów, Poland; 2 Institute of Dendrology, Polish Academy of Sciences, Parkowa 5, 62-035 Kórnik, Poland

**Keywords:** Ethnomycology, Edible mushrooms, Fungi, Mycophilia, Mycophilic, Mycophilous

## Abstract

**Background:**

The paper presents documentation of the traditional use of wild edible mushrooms in Mazovia (33,900 km^2^), a region of Poland.

**Methods:**

A total of 695 semi-structured interviews were carried out among local informants in 38 localities proportionally distributed throughout the study area (one locality approximately every 30 km), asking which mushrooms they collected and how. The species utilized were identified using visual props, morphological identification of voucher specimens, and DNA barcoding.

**Results:**

Altogether, 92 taxa identified to the species or genus level were recorded, among them 76 species used as food, 21 taxa known as toxic, and 11 taxa used for non-culinary purposes. Out of 76 identified edible fungi species, 47% (36 species) were identified using ITS DNA barcode method. Eleven of them were identified exclusively by molecular analysis. The mean number of edible taxa mentioned per interview was 9.5. Two species new to the mycobiota of Poland, *Hydnum ellipsosporum* and *Paxillus cuprinus*, were found. Frequent interaction with mushroom collectors enabled the transcription of local folk taxonomy into proper taxonomic classification and the definition of changes in local preferences concerning wild fungi collection.

**Conclusions:**

The list of species utilized is the longest regional list of edible mushrooms ever recorded during ethnomycological field research, putting the inhabitants of the studied region at the top of the mycophilia spectrum.

## Introduction

Human societies vary greatly in their frequency of utilizing fungi as food. Those which traditionally have positive attitudes towards mushroom collection and consumption are considered mycophillic, in contrast to mycophobic places where mushrooms are avoided [1]. Moreover, some mycophillic communities consider selected species of wild fungi as more valuable sources of food than wild edible plants [2, 3].

Mycophilic areas include large parts of southern and eastern Europe, Turkey, parts of Africa, Mexico, and most of Asia [4]. Traditional knowledge of fungi collection is still not well documented in many parts of the world, including major centers of mycophilia. Moreover, few studies are based on thorough ethnomycological field research. Most are focused on small communities and are sometimes based on unspecified or heterogeneous methodologies [4, 5]. Only a few studies characterize territories with large surface areas (e.g., [6–9]), and none of the abovementioned studies have attempted to conduct research that was evenly distributed over the whole studied area. Some studies were conducted only in markets or with previously selected respondents, such as mushroom vendors or people connected to mushroom commerce (e.g., [6]), which can significantly distort the overall view of community knowledge about wild growing fungi.

Prime examples of mycophilic societies are the northern Slavic nations. Valentina Wasson, one of the creators of this term, was Russian herself [1]. Actually, all northern Slavic countries (Poland, Czechia, Slovakia, Ukraine, Belarus, and Russia) and nations, respectively, display a high degree of mycophilia. In spite of this, modern ethnomycological studies documented by voucher specimens are very scarce from this area, restricted to an open air market study in south-eastern Poland [10] and a field study of Ukrainians in Romania [3]. However, the great traditions of Polish mycophilia have not gone unnoticed by ethnographers. Jerzy Wojciech Szulczewski from Poznań is the author of the first study of fungi sold in city markets in the world [11]. The use of fungi was also documented by Józef Gajek’s Polish Ethnographic Atlas team in 1964–1969 during a systematic study from 330 localities throughout Poland. This was later supplemented by further interviews. Little of this data has been published, apart from distribution maps of the use of selected species from the genera *Lactarius* and *Russula* [12]. Some archival data on the use of edible mushrooms are also available [13, 14].

Although mushrooms are eagerly collected across the whole area of Poland, our preliminary observations from one locality in this region [15] showed that the central-eastern part of Poland, within the historical region of Mazovia, displays the largest number of fungi taxa collected. Thus, we designed a study which aimed to document the use of wild edible fungi in a large area, covering the whole region, based on a large number of interviews.

Ethnomycological studies pose many problems in identification of the species listed by informants. Fruiting bodies occur only seasonally, and identification to species level is sometimes difficult even for taxonomists. DNA barcoding facilitates ethnomycological research in many ways. For example, it enables a more exact identification often only from fragments of dried mushrooms collected by the interviewees and enables proper identification of voucher specimens collected during village walks and validation of the initial identification conducted by the researcher. Unfortunately, it is still not widely used in ethnomycology as a tool to eliminate possible errors related to species identification [11, 16, 17].

The main objective of this research is to create the complete ethnomycological documentation of an entire European region with evenly distributed intensity of fieldwork throughout the entire research area. It is connected with further objectives such as:

- Finding rare and protected fungi species used among people living in the Mazovia region;
- Creating a list of locally collected fungi species list with a description of their uses;
- Creating a list of species regarded as inedible or poisonous;
- Assigning proper taxonomic nomenclature to local fungi names;
- Determining folk views on the connections between particular taxa;
- Determining the cultural salience of particular fungi taxa; and
- Detecting changes in preferences concerning wild fungi collection.

## Methods

### Study area

**Fig. 1:**
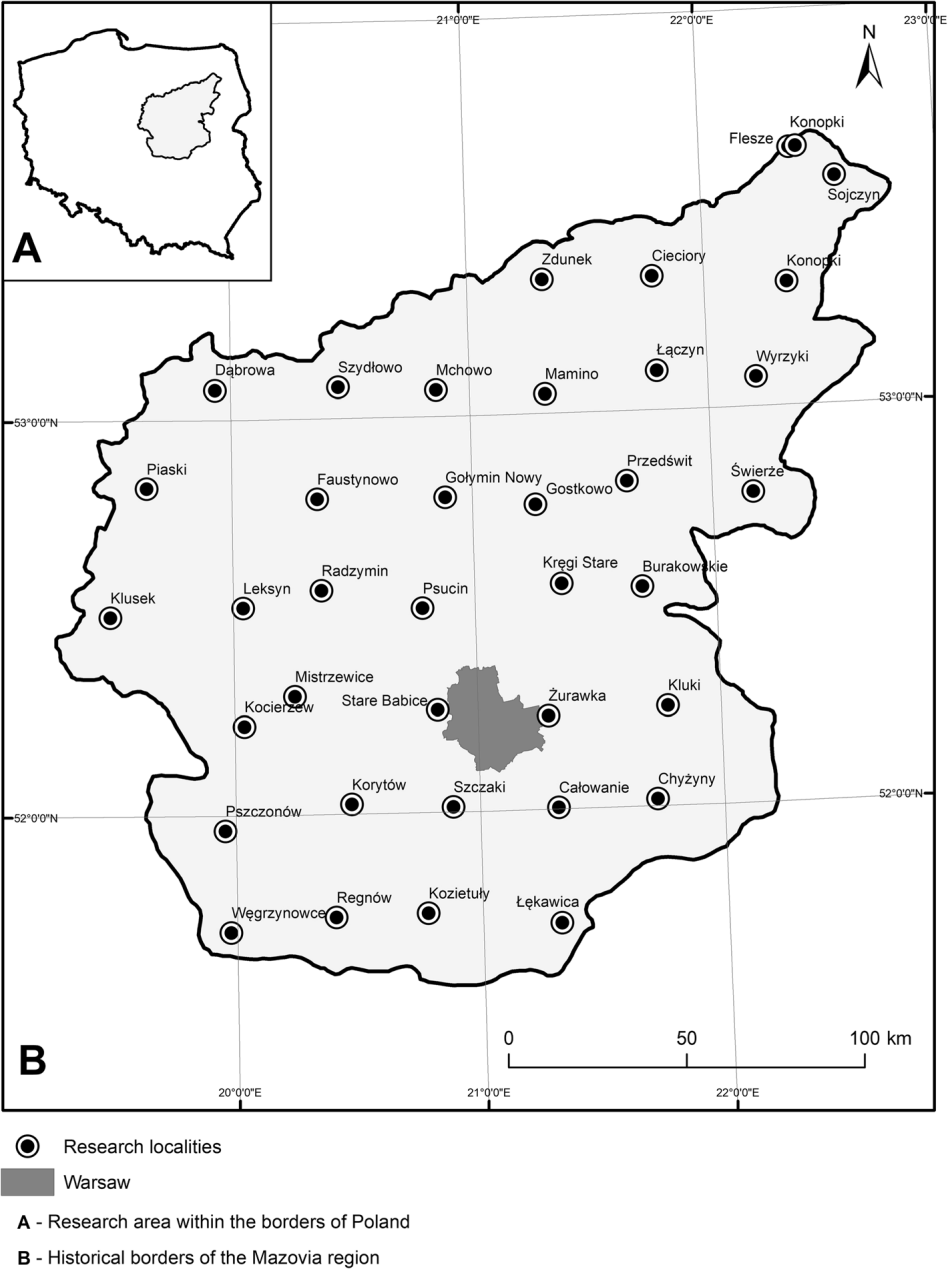
Study area

Mazovia is one of the ten major Polish historical regions within the area of present-day Poland. Throughout a major part of Polish medieval history, Mazovia was an independent principality. It consists of lands which have been united over the centuries by shared history, culture, and politics, regardless of the current administrative borders [18]. In the case of the present research, the borders of the region were based on the map created for the Historical Atlas of Poland *Mazovia in the second half of XVI century* written by Pałucki [19]. The sixteenth century borders are accepted as the best determinants of this region’s shape and are presently used as reference points during the research conducted within its area [20, 21] (Fig. 1).

The region lies mainly within the current borders of the Mazovian Voivodeship; however, its lands extend to part of the Podlasie Voivodeship in the north-east and the Łódź Voivodeship in the south-west. It spreads over the Mazovian Lowland in the valleys of the Vistula, Narew, and Bug rivers. The whole area of this region covers about 33,900 km^2^, and it is inhabited by 5.03 million people, making up 13.1% of the total population of the country [22]. The climate of Mazovia is cold temperate and has a transitional character between oceanic and continental, with high annual temperature amplitudes [23]. The average temperature in summer (VI–VIII) is about 18 °C and in winter (XII–II) − 1 °C. Average annual rainfall varies from 550 to 600 mm [24]. Forest vegetation covers 23.3% of the studied area [25]. The majority of these forests (64%) are coniferous, composed mainly of *Pinus sylvestris* (Scots pine). The other most abundant species in deciduous and mixed forests are *Quercus robur* (Pedunculate oak) and *Betula pendula* (silver birch).

It is currently difficult to find any shared cultural characteristics for people living in this historical region, but it is still inhabited by a few ethnographic groups which can be distinguished by their local cultures and traditions. These are the Kurpie, Łowiczanie, Mazurzy, and Podlasianie [20]. The capital city of Warszawa (Warsaw) is located in the center of Mazovia. In spite of the large urban sprawl around Warsaw, forests are present even in the city’s agglomeration and mushroom picking is very popular.

The research was carried out in 38 villages or small market towns which were dispersed evenly in a 30-km grid throughout the whole Mazovian region (Fig. 1). These were Burakowskie, Całowanie, Chyżyny, Cieciory, Dąbrowa, Faustynowo, Flesze, Gostkowo, Kluki, Klusek, Kocierzew, Konopki (Grajewo County), Konopki (Łomża County), Korytów, Kozietuły, Kręgi, Leksyn, Łątczyn, Łękawica, Mamino, Mchowo, Mistrzewice, Nowy Gołymin, Piaski, Przedświt, Psucin, Pszczonów, Radzymin, Regnów, Sojczyn, Stare Babice, Szczaki, Szydłowo, Świerże, Węgrzynowice, Wyrzyki, Zdunek, and Żurawka (currently the district of Sulejówek). This network of settlements forms part of the larger network of the Ethnographic Atlas of Poland, where data was also collected on mushroom gathering in 1964–1969 [26]. At that time, the ethnographer chose “large moderately backward” settlements. We selected the same settlements in order to make a return study and assess the changes in mushroom gathering.

### Field research methods

**Fig. 2:**
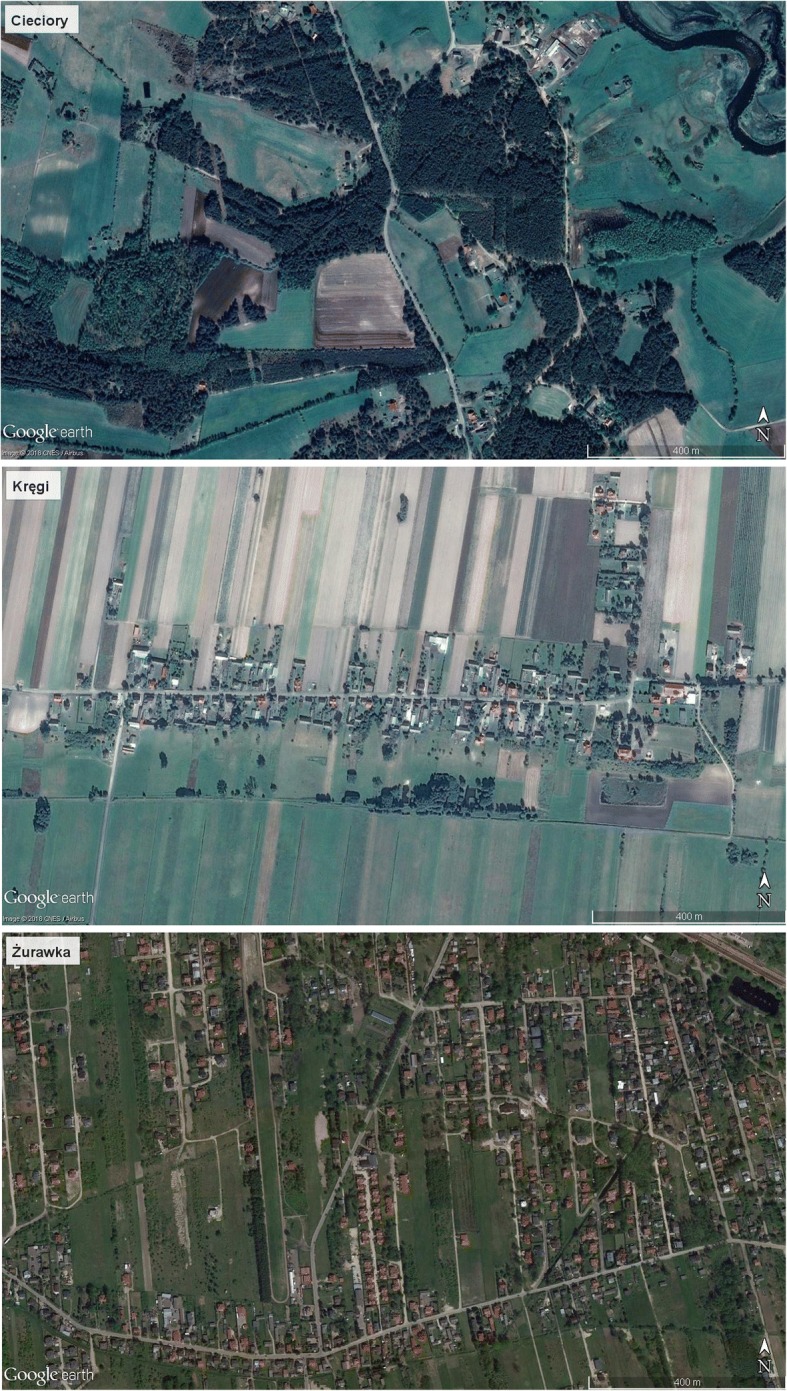
Differences in population density in selected localities. Source: Google Earth Pro

The field research took place in the months of abundance of traditionally collected wild edible fungi (IV–XI), between 2014 to 2018. Data were collected through individual semi-structured interviews with local informants, which is the classic method in ethnobiology [27]. In order to define the cultural salience of particular fungal taxa, information about macrofungi gathered or recognized as edible was collected by using the freelisting method [28, 29]. During interviews, respondents were asked separately about wild macrofungi known as edible, inedible, and used for non-consumption purposes. All freelists were made orally and written down. During interviews, the informants were also asked which species known as edible were collected currently, and which only in the past. Altogether, 695 interviews were carried out. Informants were selected during village walks or using the “snowball” sampling technique [30]. We aimed at interviewing 20 informants per locality and could not find the attempted 20 in 10 localities. These are Cieciory (10 interviews), Dąbrowa (17), Flesze (10), Konopki (Grajewo county) (10), Konopki (Łomża county) (16), Leksyn (18), Nowy Gołymin (10), Piaski (18), Wyrzyki (18), and Zdunek (8). This is connected with demographic changes which have taken place over the last five decades in some of the settlements. Since Gajek’s research, some sites that were included in the village grid have been visibly depopulated, while others have become parts of broader urbanized areas (Fig. 2).

Among the 695 respondents, women accounted for 52% (362) and men for 48% (333). The age of informants ranged from 17 to 95. The mean age was 63 (SD = 13.7) and median 64.

The majority of folk taxa listed during interviews were identified with the support of mushroom identification guides or pictures. Some of these interviews were conducted during (or soon after) mushroom collection, which enabled us to recognize taxa on the spot and to acquire voucher specimens, whose identification was later verified with DNA barcoding [31, 32]. There was at least one landscape walk or joined collection trip in each village. The majority of voucher specimens were collected fresh during field interviews, and some were acquired in dried form from respondents. The fresh specimens were dried soon after collection following the guidelines of accepted methods for DNA barcoding of fungi [33]. Fungal DNA was extracted from a small part of each fruit body using a Plant and Fungi DNA Purification Kit (Eurx), following the standard protocol. The PCR cocktail consisted of 4 ml DNA extract, 0.5 ml each of the primers (ITS5 and ITS4 in 10 nmol concentration) and 5 ml Type-it Microsatellite PCR Kit (Qiagen). PCR was carried out using the following thermocycling conditions: an initial 15 min at 95 °C, followed by 35 cycles at 95 °C for 30 s, 55 °C for 30 s, 72 °C for 1 min, and a final cycle of 10 min at 72 °C. PCR products were estimated by running a 5-ml DNA amplicon on 1.5% agarose gel for 30 min. The PCR products were sequenced using ITS4 primers at the Laboratory of Molecular Biology of Adam Mickiewicz University (Poznań). The obtained sequences were verified visually on chromatograms using BIOEDIT. Nuclear ITS sequences obtained in this study are deposited in GenBank [34], with the accession numbers listed in Table 4. Fungi names follow Index Fungorum [35]. The results were evaluated statistically using Statistica version 12.5.

### Data analysis

**Fig. 3:**
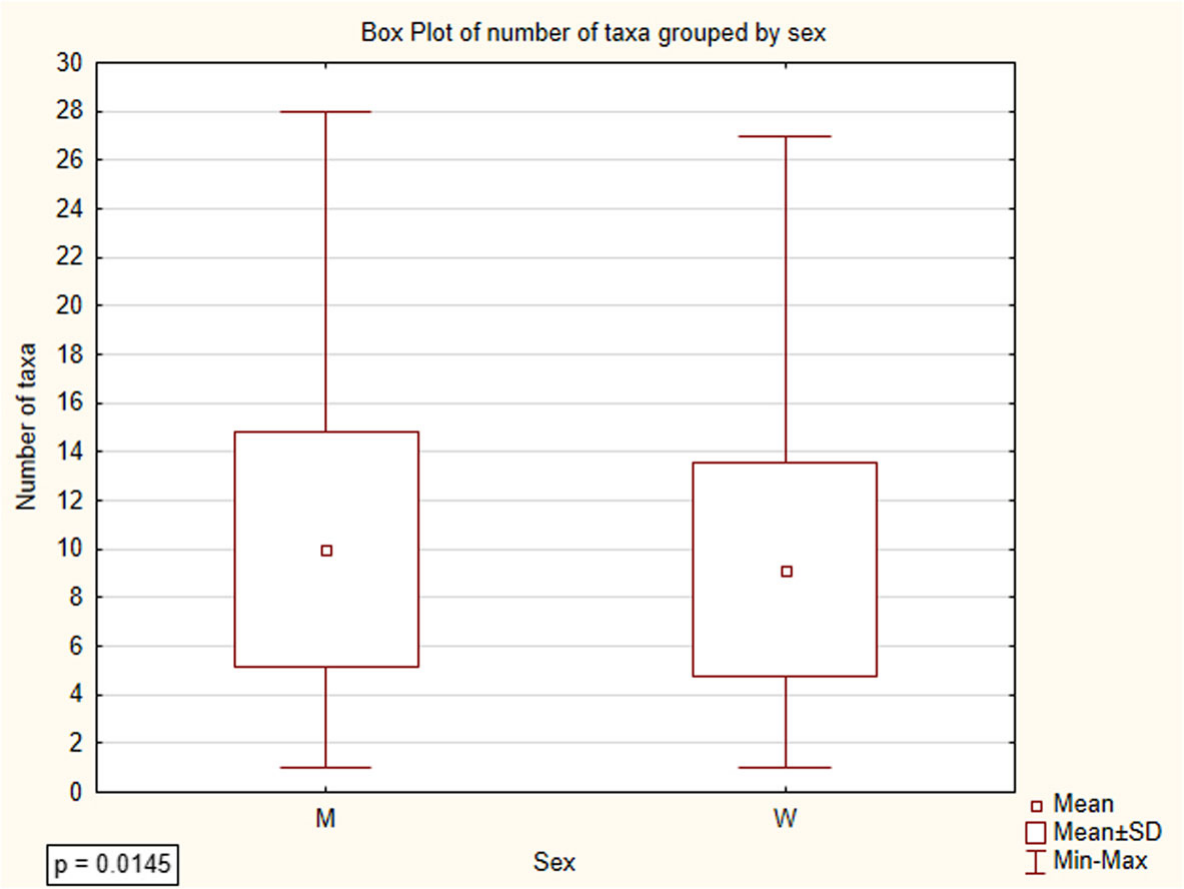
Difference between men and women in relation to knowledge about wild edible fungi. M men, W women

**Fig. 4:**
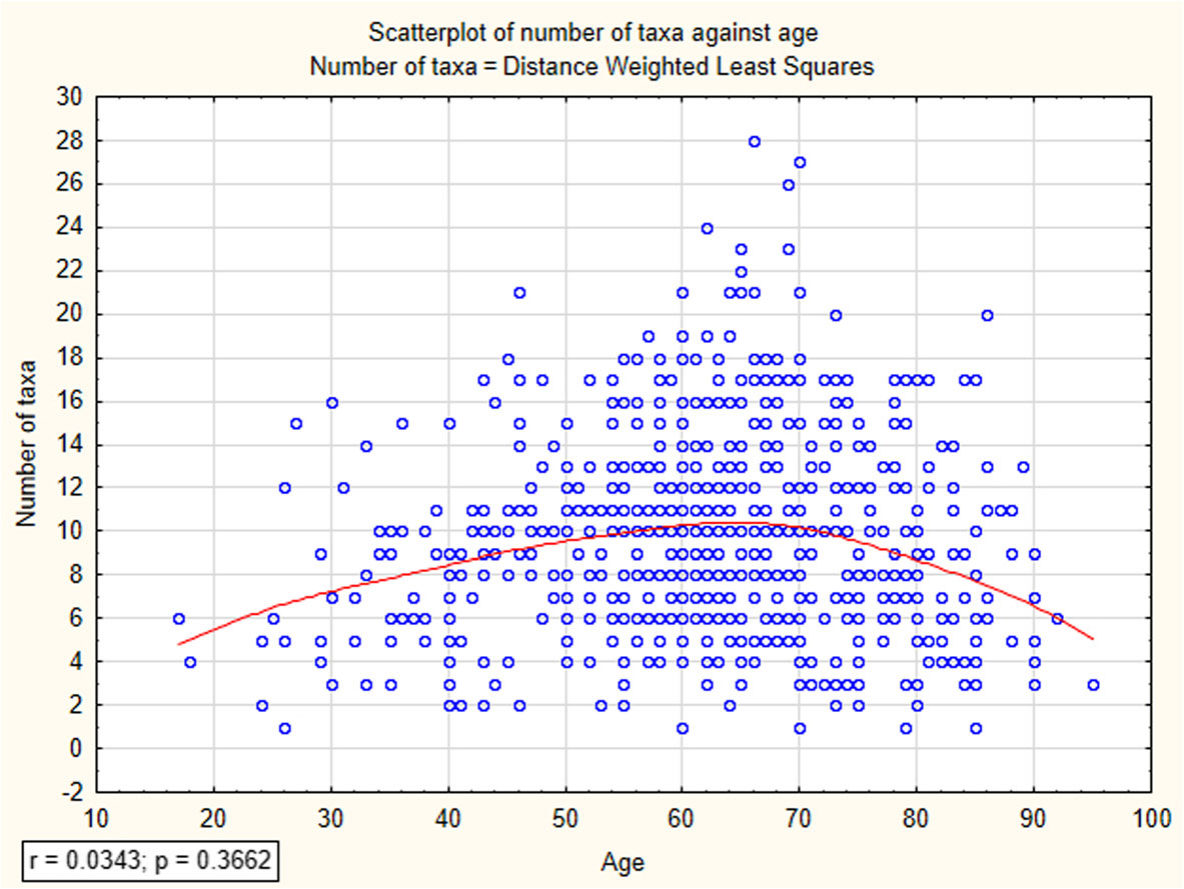
Relationship between wild edible fungi knowledge and the age of the informants

In order to measure the cultural importance of particular fungi taxa, we used Smith’s Salience Index [36]. Salience was derived using a salience index (Smith’s *S*) defined as *S* = ((*L* − *R*_*j*_ + 1)/*L*)/*N*, where *L* is the length of each list, *R*_*j*_ is the rank of item *j* in the list, and *N* is the number of lists in the sample (Tables 1, 2, and 3). The significance of differences in local knowledge about wild edible fungi between men and women was determined using the *T* test for independent samples (Fig. 3). The relation between age and local knowledge about edible fungi was analyzed by distance-weighted least squares regression (Fig. 4). Statistica version 12.5 programme was used to perform most of the statistical analyses, apart from Salience index, which was calculated from the basic data spreadsheet in Excel.

**Table 1:** Scientific and local names of fungi used for culinary purposes in Mazovia with their salience and frequency

Scientific names of folk taxa		Smith’s *S*	Frequency *n* = 695	Local names
*Agaricus* sp., mainly: *Agaricus campestris* s.l. L. including:		0.2922	228	*mainly*: pieczarka; *also*: dzika pieczarka, pieczarka łąkowa, pieczarka polna
*Agaricus arvensis* Schaeff.		0.0036	3	pieczarka leśna
*Amanita fulva* s.l. Fr		0.0327	27	czubajka, czubek, panienka, wyskoczek, żydówka
*Amanita muscaria* (L.) Lam.		0.0073	6	muchomor, muchomor czerwony
*Armillaria mellea* s.l. (Vahl) P. Kumm. including: *Armillaria borealis* Marxm. & Korhonen, *Armillariia gallica* Marxm. & Romagn.		0.3219	255	*mainly*: opieńka; *also*: opieniek, opieńka żółta, podpieniek, podpieńka, podpinka
*Armillaria gallica* Marxm. & Romagn.		0.0012	1	opieńka płowa
*Boletus edulis* s.l. Bull. (and, much more rarely, *Gyroporus castaneus* (Bull.) Quél.)		0.9157	649	*mainly*: prawdziwek, borowik; *also*: borowik szlachetny, grzyb prawy, prawdziwy, prawdziwy grzyb, prawiak, prawus, prawuszek, prawy
These species are sometimes differentiated: *Boletus reticulatus* Schaeff.		0.022	18	prawdziwek, prawdziwek biały, prawdziwek dębowy, prawdziwek jasny, prawdziwy dębowy
*Gyroporus castaneus* (Bull.) Quél.		0.0014	1	Prawdziwek piaskowiec
*Boletus subtomentosus* s.l. L. also: *Boletus ferrugineus* Schaeff. *Xerocomellus cisalpinus* (Simonini, H. Ladurner & Peintner) Klofac *Xerocomellus pruinatus* (Fr. & Hök) Šutara		0.1600	125	podgrzybnica, podgrzybniczka, zając, zajączek, zajęczak
*Calocybe/Lepista/Tricholoma* sp. including:		0.4890	361	cyz, gąski, pecłonka, prośnianka
*Tricholoma equestre* (L.) P. Kumm.		0.3230	251	*mainly*: gąska zielona, prośnianka zielona; *also*: gąska zielonka, gąska żółta, pecłonka zielona, prośnianka zielonkawa, prośnianka żółta, zielonka
*Tricholoma portentosum* (Fr.) Quel.		0.2967	231	*mainly*: gąska siwa, prośnianka siwa; *also*: gąska ciemna, gąska szara, pecłonka szara, podzielonka, prośnianka seledynowa, prośnianka szara, siwka
*Calocybe gambosa* (Fr.) Donk		0.0039	3	gąska biała
*Lepista nuda* (Bull.) Cooke		0.0024	2	gąska fioletowa, gąsówka naga
*Calvatia gigantea* (Batsch) Lloyd		0.0073	5	bździucha, purchawa, purchawiec
*Lycoperdon* sp. including: *Lycoperdon lividum* Pers.		0.0012	1	purchawa, pafbol
*Cantharellus cibarius* s.l. Fr.		0.7387	539	*mainly*: kurka, gąska; *also*: drzewiak, gąska, kurek, kurka, lisiczka
*Coprinus comatus* (O.F. Müll.) Pers.		0.0014	1	kania
*Cortinarius caperatus* (Pers.) Fr.		0.0714	61	kołpak, niemka, płachcianka, turek
*Cortinarius mucosus* (Bull.) J. Kickx		0.0012	1	tłuszczka
*Craterellus cornucopioides* (L.) Pers.		0.0156	13	cholewa, cholewka, czarna kurka, fioletowa trąba
*Gyromitra esculenta* (Pers.) Fr.		0.0643	48	babie uszy, piestrzenica
*Gyroporus cyanescens* (Bull.) Quél.		0.0721	58	*mainly*: siniak, modrzak; *also*: modrak, modrzewiak, piasecznik, piaskowiec
*Hydnum repandum* s.l. L. including: *Hydnum ellipsosporum* Ostrow & Beenken		0.0046	4	kolczak, sarenka
*Hygrophorus hypothejus* (Fr.) Fr.		0.033	25	cienka łydka, listopadka, listopadówka, przylaszczka, tłuszczka
*Imleria badia* (Fr.) Fr.		0.7959	572	*mainly*: podgrzybek; *also*: czarny łepek, podgrzyb, podgrzybek brązowy, podgrzybka, podgrzybnica, podprawdziwek, półgrzybek, półprawdziwek, siniak
*Laccaria amethystina* (Huds.) Cooke		0.0013	1	tatarka
*Lactarius deliciosus* s.l. (L.) Pers.		0.3115	242	rydz
*Lactarius deterrimus* Gröger		0.0026	2	rydz żółty
*Lactarius piperatus* (L.) Pers.		0.0046	4	bil, bily, mleczak
*Lactarius vellereus* (Fr.) Fr.		0.0069	6	chrząszcz, gruzd, kobyłka
*Lactarius volemus* (Fr.) Fr.		0.0149	13	dójka, krowa, krówka, krówski rydz
*Leccinum* sp. including:		0.347	252	kowale, kozaki, kozery, kozyrki, koźlaki, koźlary, koźlarze
*Leccinum aurantiacum* s.l. (Bull.) Gray		0.5368	397	*mainly*: osak; *also*: czerwona główka, czerwoniak, czerwoniak bordowy, czerwonogłowiec, czerwonołepek, czerwony, czerwony łepek, kowalik, kozak czerwony, kozer czerwony, koźlak czerwony, koźlar czerwony, koźlarz czerwony, krawiec, Lesiak, olszak, olszyn, osiniak, pamfil, pociech, pociecha, stołyngwa, zapałka
From *L. aurantiacum*, the following species are sometimes differentiated: *Leccinum quercinum* (Pilát) E.E. Green & Watling		0.0081	6	dębniak
*Leccinum versipelle* (Fr. & Hök) Snell		0.004	3	czerwoniak, czerwoniak jasny
*Leccinum vulpinum* Watling		0.0038	3	koźlarz brązowy, osak brązowy, osak ciemnobrązowy
Brown-capped species, mainly *Leccinum scabrum* s.l. (Bull.) Gray, also *L. pseudoscabrum* (Kallenb.) Mikšik and *L. variicolor* Watling		0.502	365	*mainly*: kozak szary; *also*: baba, brzeźniak, brzozowiak, kowal siwy, kozaczek, kozak, kozak brązowy, kozak siny, kozak siwy, kozer, kozerek siwy, kozioł, koziołek, koźlak, koźlak biały, koźlak brązodwy, koźlak jasny, koźlak siwy, koźlak szary, koźlak szary, koźlar brązowy, koźlar siwy, koźlar szary, koźlarek, koźlarz, koźlarz ciemny, koźlarz siwy, koźlarz szary, podbrzeźniak, siwek
Sometimes differentiated: *L. pseudoscabrum* (Kallenb.) Mikšik		0.0163	14	koziołek czarny, koźlak ciemno-szary, koźlak czarny, koźlar ciemny, koźlar czarny
*Macrolepiota procera* s.l. (Scop.) Singer (most often), occasionally also: *Chlorophyllum* sp. including: *Chlorophyllum brunneum* (Farl. & Burt) Vellinga *Chlorophyllum olivieri* (Barla) Vellinga *Chlorophyllum rhacodes* (Vittad.) Vellinga		0.4195	323	*mainly*: kania; *also*: baran, czubajka, czubak, drapka, gapa, kania polna, sowa
*Chlorophyllum rhacodes* (Vittad.) Vellinga (sometimes distinguished from *Macrolepiota*)		0.0036	3	kania czerwieniejąca, kania leśna
*Marasmius oreades* (Bolton) Fr.		0.1068	79	*mainly*: przydróżka, psiak, twardzioszek, tańcowniczka; *also*: gromadka, murawka, podróżniak, podróżniczek, podróżnik, przydrożniak, przydróżniczek, psi grzyb, rzędówka, tanecznik, tanieczniczka, tańcownica, tańcownik, tątka, toneczniczka, tonka, twardzioszek przydrożny, wysrandek, wysranek, wysrojdek, wywieruszka, zawieruszka
*Morchella* sp*.* mainly *Morchella esculenta* (L.) Pers. and *Morchella conica* s.l Pers.		0.0316	27	smardz, smarż
*Neoboletus luridiformis* (Rostk.) G. Wu & Zhu L. Yang		0.0013	1	pójdziec
*Paxillus involutus* s.l. (Batsch) Fr. including: *Paxillus cuprinus* Jargeat, Gryta, J.-P. Chaumeton & Vizzini		0.3149	264	olchówka, olszówka
*Pleurotus ostreatus* s.l. (Jacq.) P. Kumm. including: *Pleurotus cornucopiae* (Paulet) Rolland		0.0148	12	boczniak
*Ramaria* sp.		0.0138	12	koralówka, kozia broda, kozia bródka
*Russula* sp.		0.1639	134	betka, gołąbek, serojeżka, serowiatka, surojadka, surojeżka, surowiatka, syrowiatka
Grayish species (mainly *Russula aeruginea* s.l Lindbl. ex Fr.) and green ones (*R. virescens* (Schaeff.) Fr.)		0.1378	113	*mainly*: gołąbek, betka siwa, betka zielona; *also*: gołąbek biały, gołąbek siwy, gołąbek szary, gołąbek zielony, serowiatka siwa, siwek, surojadka szara, surojadka zielona, surowiatka biała, surowiatka siwa
Sometimes differentiated: *Russula virescens* (Schaeff.) Fr.		0.0219	18	betka zielona, gołąbek, gołąbek zielony
Reddish species including: *Russula integra* (L.) Fr. *Russula nitida* (Pers.) Fr. *Russula alutacea* (Fr.) Fr.		0.0605	48	*mainly*: betka, surowiadka, gołąbek czerwony; *also*: betka czerwona, betka różowa, cukrówka, gołąbek bordowy, maślanka czerwona, serowiatka różowa, surojadka, surojadka czerwona, surowiatka czerwona, surowiatka różowa, syrowiatka
*Russula nigricans* Fr.		0.0135	12	świnka
Yellow-capped species, including: *Russula ochroleuca* Fr. *Russula claroflava* Grove		0.0246	21	betka pomarańczowa, betka żółta, gołąbek żółty, maślanka żółta, maślanka, serowiatka żółta, surowiatka żółta
*Sarcodon squamosus* s.l. (Schaeff.) Quél.		0.0997	82	*mainly*: sarna, krowia morda; *also*: bycze serce, krowia gęba, sarenka, wola morda, woli morda, wołowy język
*Scleroderma citrinum* Pers.		0.0083	7	bycze jajka, tęgoskór, trufla
*Sparassis crispa* (Wulf.) Fr.		0.0083	7	jarosz, kozia broda
*Suillus luteus* (L.) Roussel (mainly) and other *Suillus* spp. including:		0.702	521	maślak, maśluk, pampek, pępek, ślimak
*Suillus bovinus* (L.) Roussel		0.0712	58	*mainly*: sitarz, sitak; *also*: maślak sitarz, podgrzybek sitarz, sitarek, sitawka, sitek, sitka, sitowiak
*Suillus granulatus* (L.) Roussel		0.0037	3	maślak jasny, maślak wczesny
*Suillus grevillei* (Klotzsch) Singer		0.0162	14	maślak modrzewiowy, maśluk modrzewiowy, modrzewiak
*Suillus variegatus* (Sw.) Richon & Roze		0.0811	68	*mainly*: bagniak, jakubek, *also*: błotniak, błotniczek, lesiak, miodówka, miodziak, podgrzybek żółty, twardak, twardziak
*Tricholomopsis rutilans* (Schaeff.) Singer		0.0025	2	rycerzyk, tłuściocha
*Tuber* sp. P. Micheli ex F.H. Wigg.		0.0049	3	trufla
*Tylopilus felleus* (Bull.) P. Karst.		0.0012	1	szatan

**Table 2:** Scientific and local names of toxic and inedible fungi known in Mazovia with their salience and frequency

Scientific names of folk taxa		Smith’s *S*	Frequency, *n* = 695	Local names
Other very small *Agaricales* regarded as toxic or worthless		0.0423	31	*mainly*: psiak; *also*: blaszak, blaszkowaty, psi, psia betka, psi grzyb, psio betka, psiuch
*Agaricus xanthodermus* Genev.		0.0013	1	trująca pieczarka
*Amanita* sp. including:		0.4804	336	muchary, muchomory
Species with spotted cups, mainly *Amanita muscaria* (L.) Lam.		0.3048	219	*mainly*: muchomor, muchomor czerwony; *also*: muchomor muchar, muchar czerwony, muchomor, muchomor kropkowaty, muchomor pospolity, muchomor pstry
The following species is sometimes differentiated: *Amanita pantherina* (DC.) Krombh.		0.0025	2	muchomor plamisty
Species with not-spotted caps, mainly *Amanita phalloides* s.l. Vaill. ex Fr.		0.2767	200	*mainly*: muchomor sromotnikowy, sromotnik; *also*: muchar siwy, muchar sromotnik, muchomor biały, muchomor czubiasty, muchomor siwy, muchomor sromotnik, muchomor sromotny, muchomor sromotny, muchomor szary, muchomor zielonkawy, muchomor zielony, sromotniak
The following species are sometimes differentiated:				
*Amanita citrina* Pers.		0.0122	9	muchomor cytrynowy, muchomor żółty
*Amanita virosa* s.l. Bertill.		0.0122	16	muchar białawy, muchomor biały
*Armillaria* sp. (Fr.) Staude		0.0042	3	opieńka
*Calvatia*, *Bovista*, or *Lycoperdon* sp.		0.0094	7	bycze jaja
*Chlorophyllum rhacodes* s.l. (Vittad.) Vellinga		0.0096	7	kania, trująca kania
*Coprinopsis* sp. P. Karst		0.0014	1	czernidłak
*Galerina marginata* (Batsch) Kühner		0.0013	1	hełmówka jadowita
*Hygrophoropsis aurantiaca* (Wulfen) Maire		0.0109	8	fałszywa gąska, fałszywa kurka, pieprznik jadowity, trująca kurka
*Lactarius* sp. including:		0.0052	4	mleczaki
*Lactarius aurantiacus* (Pers.) Gray		0.0012	1	mleczaj gorzki
*Lactarius piperatus* (L.) Pers.		0.0013	1	bil
*Lactarius torminosus* (Schaeff.) Gray		0.0053	4	trująca krowia morda, trujący rydz, wełnianka
*Paxillus involutus* s.l. (Batsch) Fr.		0.0452	33	*mainly*: olszówka; *also*: krowiak, świńska olszówka
*Tapinella atrotomentosa* (Batsch) Šutara		0.0081	6	krzywogęba, krowia gęba, świnia, włochata olszówka
Bracket fungi (*Polyporales* spp.) in general		0.004	3	huby
*Ramaria* sp.		0.0013	1	kozia bródka
*Rubroboletus satanas (*Lenz) Kuan Zhao & Zhu L. Yang		0.0071	5	borowik szatan, szatanista, borowik szatański
*Russula* sp. including:		0.0109	8	betki, surowiatki
*Russula emetica* (Schaeff.) Pers.		0.0137	10	betka czerwona, trujący gołąbek, surowiatka, gołąbek czerwony, surowiatka trująca, czerwona siwka
*Russula fellea* (Fr.) Fr.		0.0027	2	betka żółta
*Tylopilus felleus* (Bull.) P. Karst.		0.3666	264	*mainly*: szatan, goryczak; *also*: goryczak, gorzkal, gorzkelec, gorzki, gorzkowiec, gorzkówka, goszkielec, goszniak, gosztelec, piołun gorzkowiec, podgorzelec, prawdziwek szatan, prawdziwek trujak, szatan, szatan podgrzybek, świnia, zając

**Table 3:** Scientific and local names of other useful fungi known in Mazovia with their salience and frequency

Scientific names of folk taxa		Smith’s *S*	Frequency, *n* = 695	Use	Local names
*Amanita muscaria* (L.) Lam.		0.0934	66	fly trap, psychoactive	Table 2
*Boletus edulis* Bull.		0.0015	1	dye	Table 1
*Claviceps purpurea* (Fr.) Tul.		0.0015	1	abortifacient	sporysz
*Gyromitra esculenta* (Pers.) Fr.		0.0028	3	medicine	Table 1
*Polyporales* sp.				decoration, medicine	Table 2
*Piptoporus betulinus* (Bull.) B.K. Cui, M.L. Han & Y.C. Dai		0.0161	11	medicine, decoration	biała huba, huba brzozowa
*Inonotus obliquus* (Ach. ex Pers.) Pilát		0.0015	1	medicine	czarna huba, huba brzozowa
*Psilocybe* sp. (Fr.) P. Kumm.		0.0085	8	psychoactive	grzybek, grzybek halucynek, halucynek, łysiczka
*Rubroboletus satanas* (Lenz) Kuan Zhao & Zhu L. Yang		0.0015	1	fly trap	Table 2
*Scleroderma citrinum* Pers.		0.0015	1	fly trap	Table 1
*Suillus luteus* (L.) Roussel		0.0015	1	axle grease	Table 1
*Tapinella atromentosa* (Batsch) Šutara		0.0015	1	fly trap	Table 2

In order to compare our results with those obtained between 1964 and 1969 by Józef Gajek’s team, we analyzed 48 questionnaires gathered by the team in selected Mazovian villages (there were 38 localities but in some places the research was repeated). During the analysis, we excluded questionnaires with data collected without using the freelisting method and also questionnaires with visible identification errors. Questionnaires with data acquired without using the freelisting method were characterized by the order of listed species, which coincided with the order of species listed in the mushroom guide written by Henryk Orłoś in 1963 [37]. It is known that this guide was used as a support for species identification during Gajek’s research. Determination of obvious identification errors was possible due to the very long local fungi name list created during present research in the same villages. In a few cases, popular local names were assigned to the guide’s illustrations depicting rare or locally absent species with characteristics similar to those of commonly collected and abundant species.

## Results and discussion

### General information

During field research, we recorded the use of 65 fungi folk taxa which were listed as edible. In these folk taxa, we identified 76 scientific taxa on the genus or species (Table 1). We identified 21 taxa of species considered as inedible or poisonous to the genus or species level and 3 folk taxa on levels higher than family (Table 2). We also recorded the uses of 11 fungi species or genera for other purposes than food (i.e., medicinal and hallucinogenic, Table 3). Bearing in mind that recorded folk taxa correspond to different taxonomic ranks such as genera or orders, these folk classifications can actually apply to dozens of other different scientific species, which are rare (and rarely used) but similar and related to popularly recognized taxa. Considering that in a few cases the same taxon was present on more than one list (i.e., edible, toxic, other), there were altogether 92 different fungi taxa identified to the genus or species level, recorded as used or known, now or in the past, by people living in Mazovia.

The mean number of recorded edible fungal taxa is 9.5 and the median is 9, minimum 1 and maximum 28 per interview. We detected a very small, but significant difference between men and women in relation to knowledge about wild edible fungi (Fig. 3; *p* = 0.0145).

According to the results, men display more diversified knowledge considering wild edible fungi than women. Men reported on average 9.9 ± 4.8 fungi taxa while women 9.1 ± 4.4. There was no significant correlation between age of respondents and number of listed edible species; however, the graph of weighted least squares regression suggests that informants aged between 60 and 70 have on average the largest knowledge of wild edible fungi (Fig. 4).

**Fig. 5:**
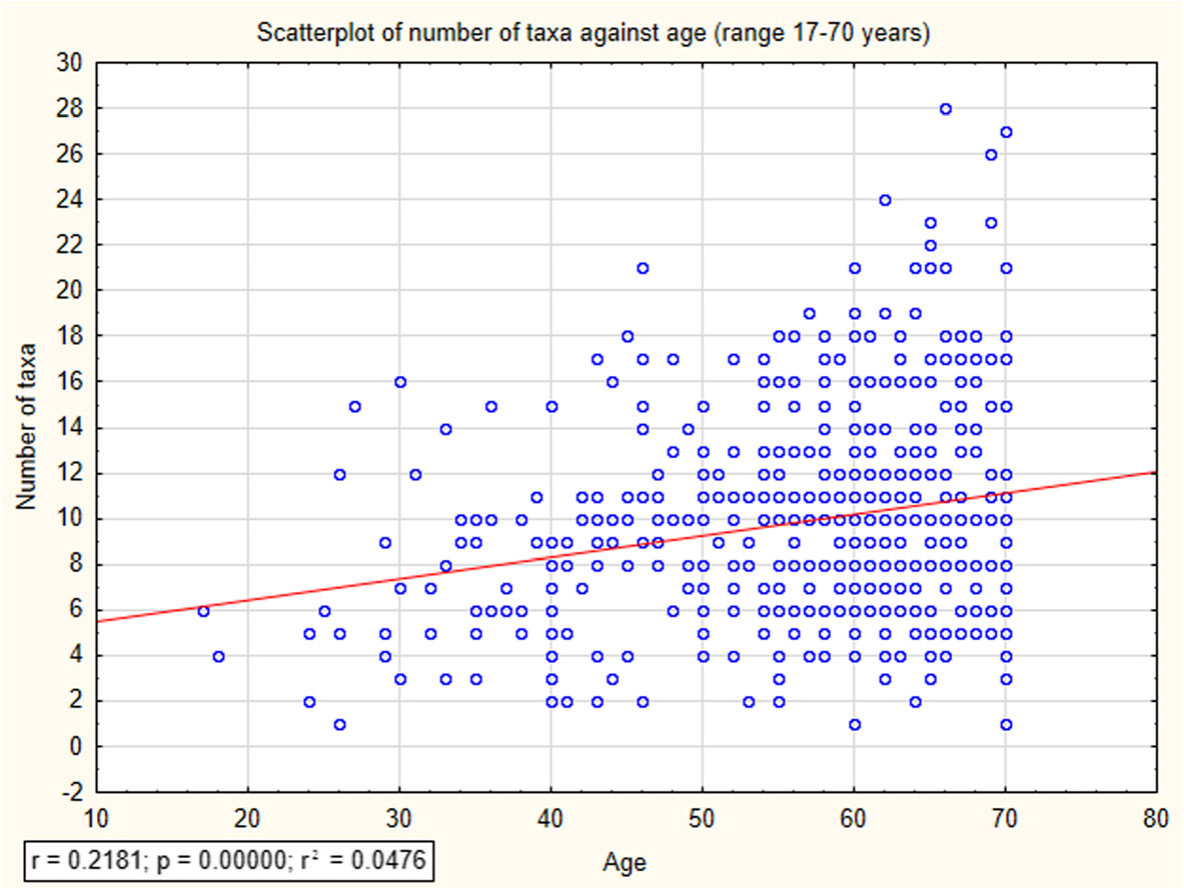
Relationship between wild edible fungi knowledge and the age of the informants ranged between 17 and 70 years

However, after removing results for ages over 70, when the cognitive capacity of informants drops, we acquired a significant correlation between these two factors (Fig. 5).

The mean number of listed inedible or poisonous fungi taxa is 1.7 (median = 2, minimum = 0, maximum = 6), and the mean number of fungi taxa with other useful properties is 0.15 (median = 0, min = 0, max = 3).

Taking into account the mean number of species listed, the largest number of fungi taxa are collected in Żurawka, Mińsk county (mean = 14.7); Faustynowo, Ciechanów county (mean = 12.75); and Węgrzynowice, Tomaszów county (mean = 12.26). When all the lists from one settlement were added together, the longest lists of edible fungi taxa were acquired for Pszczonów = 41, Żurawka = 37, Szczaki = 36, and Korytów and Węgrzynowice = 33. All these villages are situated close to each other in the central and south-western parts of the Mazovia region.

Mushrooms are frequently used in a variety of boiled and fried dishes. Many taxa are also preserved (dried, pickled, or frozen after brief boiling). The range of mushroom dishes and their processing techniques is so diverse that it is worthy of discussion in a separate paper.

### Diachronic differences

**Fig. 6:**
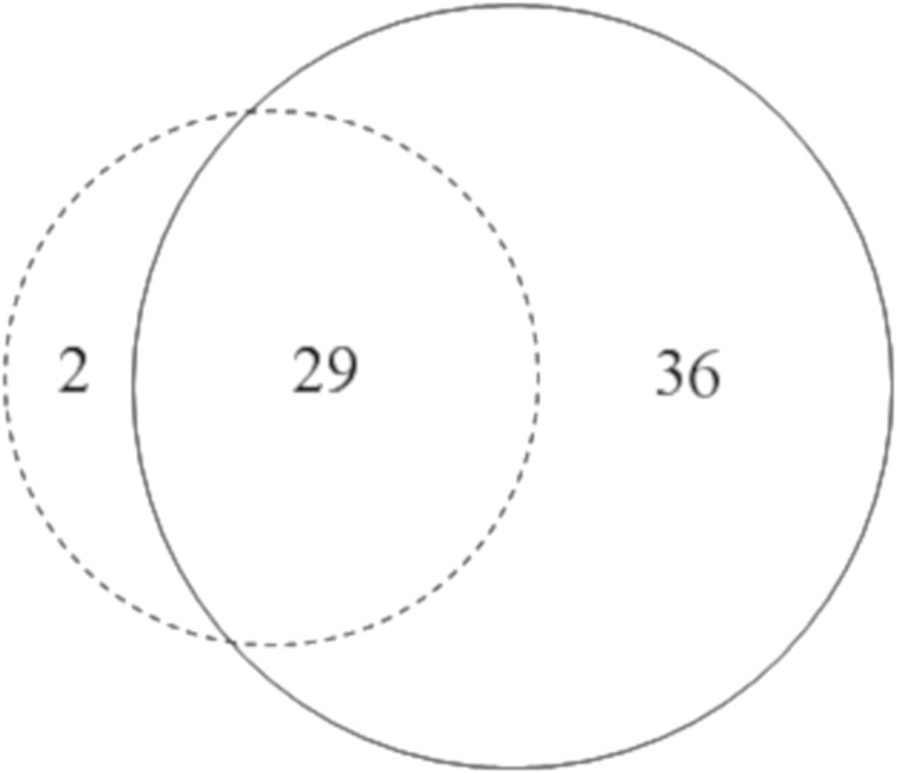
The relationships between the taxa recorded in Gajek’s questionnaire from 1964 to 1969 (dotted line) and present research from 2014 to 2018 (solid line)

In the data from the 1960s, 31 fungi folk taxa were identified as listed by Mazovian informants during Gajek’s research. In comparison, current field research based only on interviews conducted in the same localities enabled the identification of 65 wild edible fungi folk species used by Mazovian communities (after the DNA barcoding, the number of identified taxa increased to 76). Only two species present on Gajek’s list were not recorded during our research (Fig. 6). These are *Sarcodon imbricatus* and *Xerocomellus chrysenteron*. Both of them were listed in Pszczonów village. In the case of *Sarcodon imbricatus*, it is possible that it was confused during identification with *Sarcodon squamosus*, which was identified in the same village during the present research and was not present in the guide used for species identification during Gajek’s research [37]. It is still possible that this species occurs and is used there. *Xerocomellus chrysenteron*, on the other hand, is very abundant in Mazovian forests. Further DNA barcode analysis shows that *Xerocomellus* species are perceived by Mazovian residents as different variants of *Boletus subtomentosus* and are known under one collective taxa “zajączek” (Table 4). This probably also applies to *Xerocomellus chrysenteron*. However, because this species was not identified by the respondents during field research or by DNA barcode analysis of collected voucher specimens, it is not included in the present list of fungi taxa known as edible in the region.

**Table 4:** The list of voucher specimens and the results of DNA barcoding

Voucher no.	Molecular identification	Accession number	Similarity	Reference sequences	Specimen’s local name
WA0000071001	*Russula nitida* (Pers.) Fr.	MK028864	99.85	KU205349	Betka czerwona
WA0000071002	*Xerocomellus cisalpinus* (Simonini, H. Ladurner & Peintner) Klofac	MK028865	99.86	UDB002180	Zajączek
WA0000071003	*Xerocomellus cisalpinus* (Simonini, H. Ladurner & Peintner) Klofac	MK028866	100	UDB002180	Zajączek
WA0000071004	*Cantharellus cibarius* Fr.	MK028867	99.31	LC085408	Kurka
WA0000071005	*Amanita fulva* Fr.	MK028868	100	UDB002417	Panienka
WA0000071006	*Tricholoma equestre* (L.) P. Kumm.	MK028869	100	UDB011389	Gąska zielona
WA0000071007	*Russula aeruginea* Lindbl. ex Fr.	MK028870	99.84	UDB000341	Gołąbek
WA0000071008	*Russula aeruginea* Lindbl. ex Fr.	MK028871	100	UDB000341	Gołąbek
WA0000071009	*Russula aeruginea* Lindbl. ex Fr.	MK028872	100	UDB000341	Ggołąbek siwy
WA0000071010	*Leccinum versipelle* (Fr. & Hök) Snell	MK028873	99.76	UDB019772	Koźlak
WA0000071011	*Leccinum scabrum* (Bull.) Gray	a.f.	–	–	Kozak
WA0000071012	*Armillaria gallica* Marxm. & Romagn.	MK028874	99.75	KT822312	Opieńka
WA0000071013	*Boletus edulis* Bull.	MK028875	99.70	DQ131623	Prawdziwek
WA0000071014	*Boletus reticulatus* Schaeff.	MK028876	99.70	KY595992	Prawdziwek
WA0000071015	*Chlorophyllum brunneum* (Farl. & Burt) Vellinga	MK028877	99.85	AY083208	Kania
WA0000071016	*Xerocommelus cisalpinus* (Simonini, H. Ladurner & Peintner) Klofac	MK028878	99.85	UDB002180	Zajączek
WA0000071017	*Suillus bovinus* (L.) Roussel	MK028879	100	KF482482	Maślak
WA0000071018	*Suillus luteus* (L.) Roussel	MK028880	100	KX230614	Pępek
WA0000071019	*Craterellus cornucopoides* (L.) Pers.	MK028881	100	KT693262	Cholewa
WA0000071020	*Russula aeruginea* Lindbl. ex Fr.	MK028882	100	UDB000341	Gołąbek
WA0000071021	*Russula claroflava* Grove	a.f.	–	–	Gołąbek żółty
WA0000071022	*Xerocomellus cisalpinus* (Simonini, H. Ladurner & Peintner) Klofac	MK028883	99.51	UDB002180	Zajączek
WA0000071023	*Agaricus arvensis* Schaeff.	MK028884	99.51	JF797194	Pieczarka
WA0000071024	*Chlorophyllum olivieri* (Barla) Vellinga	MK028885	99.85	UDB031330	Kania czerwieniejąca
WA0000071025	*Macrolepiota procera* (Scop.) Singer	MK028886	100	UDB015607	Kania
WA0000071026	*Suillus grevillei* (Klotzsch) Singer	MK028887	100	KM085409	Maślak modrzewiowy
WA0000071027	*Gyroporus castaneus* (Bull.) Quél.	MK028888	100	UDB023475	Prawdziwek
WA0000071028	*Paxillus cuprinus*Jargeat, Gryta, J.-P. Chaumeton & Vizzini	MK028889	100	KF261422	Olszówka
WA0000071029	*Xerocomellus cisalpinus* (Simonini, H. Ladurner & Peintner) Klofac	MK028890	99.71	UDB002180	Zajączek
WA0000071030	*Hydnum ellipsosporum*Ostrow & Beenken	MK028891	100	HM189766	Kolczak
WA0000071031	*Russula nigricans* Fr.	MK028892	100	UDB000011	Świnka
WA0000071032	*Gyroporus cyanescens* (Bull.) Quél.	MK028893	100	UDB015653	Piaskowiec
WA0000071033	*Imleria badia* (Fr.) Fr.	MK028894	100	KX756408	Siniak
WA0000071034	*Cantharellus cibarius* Fr.	MK028895	99.27	KT693262	Kurka
WA0000071035	*Calocybe gambosa* (Fr.) Donk	MK028896	99.70	UDB000593	Gąska biała
WA0000071036	*Calvatia gigantea* (Batsch) Lloyd	MK028897	100	AJ617492	Purchawa
WA0000071037	*Suillus luteus* (L.) Roussel	MK028898	100	KX230614	Maślak
WA0000071038	*Leccinum pseudoscabrum* (Kallenb.) Mikšik	a.f.	–	–	Koźlak
WA0000071039	*Leccinum scabrum* (Bull.) Gray	a.f.	–	–	Kozak
WA0000071040	*Agaricus arvensis* Schaeff.	MK028899	99.57	EF460362	Pieczarka
WA0000071041	*Imleria badia* (Fr.) Fr.	MK028900	100	KX756408	Podgrzybek
WA0000071042	*Imleria badia* (Fr.) Fr.	MK028901	99.65	KX756408	Podgrzybek
WA0000071043	*Lycoperdon lividum* Pers.	MK028902	100	DQ112600	Purchawka, pafbol
WA0000071044	*Coprinus comatus* (O.F. Müll.) Pers.	a.f.	–	–	Kania
WA0000071045	*Leccinum pseudoscabrum* (Kallenb.) Mikšik	a.f.	–	–	Koźlarz ciemny
WA0000071046	*Agaricus arvensis* Schaeff.	MK028903	98.72	EF460362	Pieczarka
WA0000071047	*Boletus reticulatus* Schaeff.	MK028904	99.46	DQ131610	Prawdziwek
WA0000071048	*Boletus reticulatus* Schaeff.	MK028905	99.46	DQ131610	Prawdziwek dębowy
WA0000071049	*Boletus edulis* Bull.	MK028906	99.72	KP031595	Borowik
WA0000071050	*Boletus edulis* Bull.	MK028907	99.58	KP031595	Borowik
WA0000071051	*Leccinum aurantiacum* (Bull.) Gray	MK028908	98.94	UDB019627	Osak
WA0000071052	*Cortinarius caperatus* (Pers.) Fr.	MK028909	99.69	DQ367911	Turek
WA0000071053	*Suillus luteus* (L.) Roussel	MK028910	100	KX230614	Maślak
WA0000071054	*Boletus edulis* Bull.	a.f.	–	–	Prawdziwek
WA0000071055	*Boletus edulis* Bull.	MK028911	99.71	KX756408	Borowik
WA0000071056	*Imleria badia* (Fr.) Fr.	MK028912	99.81	KX756408	Podgrzybek
WA0000071057	*Boletus ferrugineus* Schaeff.	MK028913	99.84	UDB001674	Zając
WA0000071058	*Leccinum aurantiacum* (Bull.) Gray	MK028914	98.94	UDB011697	Osiniak
WA0000071059	*Sarcodon squamosus* (Schaeff.) Quél.	MK028915	100	UDB001707	Sarna
WA0000071060	*Boletus edulis* Bull.	MK028916	99.72	KP031595	Prawdziwek
WA0000071061	*Suillus luteus* (L.) Roussel	MK028917	100	KX230614	Maślak
WA0000071062	*Armillaria borealis* Marxm. & Korhonen	MK028918	99.75	UDB015538	Opieńka
WA0000071063	*Leccinum variicolor*Watling	MK028919	99.75	AF454572	Koźlak
WA0000071064	*Marasmius oreades*(Bolton) Fr.	MK028920	99.57	UDB017590	Tańcowniczka
WA0000071065	*Suillus luteus* (L.) Roussel	MK028921	100	KX230614	Maślak
WA0000071066	*Boletus edulis* Bull.	MK028922	100	KP031595	Prawdziwek
WA0000071067	*Imleria badia* (Fr.) Fr.	MK028923	99.82	KX756408	Podgrzybek
WA0000071068	*Imleria badia* (Fr.) Fr.	MK028924	99.82	KX756408	Podgrzybek
WA0000071069	*Boletus edulis* Bull.	MK028925	100	KP031595	Prawdziwek
WA0000071070	*Suillus bovinus* (L.) Roussel	MK028926	99.85	KF482482	Maślak
WA0000071071	*Cantharellus cibarius* Fr.	MK028927	99.31	LC085408	Kurka
WA0000071072	*Morchella esculenta* (L.) Pers.	MK028928	99.43	MF228808	Smardz
WA0000071073	*Boletus edulis* Bull.	MK028929	100	KP031595	Prawdziwek
WA0000071074	*Imleria badia* (Fr.) Fr.	MK028930	99.82	KX756408	Podgrzybek
WA0000071075	*Imleria badia* (Fr.) Fr.	MK028931	100	KX756408	Podgrzybek
WA0000071076	*Imleria badia* (Fr.) Fr.	MK028932	100	KX756408	Podgrzybek
WA0000071077	*Imleria badia* (Fr.) Fr.	MK028933	100	KX756401	Podgrzybek
WA0000071078	*Imleria badia* (Fr.) Fr.	MK028934	99.82	KX756408	Podgrzybek
WA0000071079	*Imleria badia* (Fr.) Fr.	MK028935	100	KX756401	Podgrzybek
WA0000071080	*Imleria badia* (Fr.) Fr.	a.f.	–	–	Podgrzybek
WA0000071081	*Boletus edulis* Bull.	MK028936	100	KP031595	Prawdziwek
WA0000071082	*Boletus edulis* Bull.	MK028937	100	KP031595	Prawdziwek
WA0000071083	*Imleria badia* (Fr.) Fr.	MK028938	100	KT334754	Podgrzybek
WA0000071084	*Imleria badia* (Fr.) Fr.	a.f.	–	–	Podgrzybek
WA0000071085	*Suillus luteus* (L.) Roussel	MK028939	100	KX230614	Maślak
WA0000071086	*Imleria badia* (Fr.) Fr.	MK028940	100	KX756408	Podgrzybek
WA0000071087	*Sarcodon squamosus* (Schaeff.) Quél.	MK028941	99.34	UDB001707	Krowia gęba
WA0000071088	*Suillus bovinus* (L.) Roussel	MK028942	100	KF482482	Sitak
WA0000071089	*Suillus luteus* (L.) Roussel	MK028943	100	UDB002180	Maślak
WA0000071090	*Sarcodon squamosus* (Schaeff.) Quél.	a.f.	–	–	Sarna
WA0000071091	*Xerocomellus cisalpinus* (Simonini, H. Ladurner & Peintner) Klofac	MK028944	100	KX230614	Zajączek
WA0000071092	*Suillus luteus*(L.) Roussel	MK028945	100	KX230614	Maślak
WA0000071093	*Imleria badia* (Fr.) Fr.	MK028946	100	KX756408	Podgrzybek
WA0000071094	*Boletus edulis* Bull.	MK028947	100	KP031595	Prawdziwek
WA0000071095	*Xerocomellus cisalpinus* (Simonini, H. Ladurner & Peintner) Klofac	a.f.	–	–	Zajączek
WA0000071096	*Xerocomellus cisalpinus* (Simonini, H. Ladurner & Peintner) Klofac	MK028948	100	UDB002180	Zajączek
WA0000071097	*Xerocomellus cisalpinus* (Simonini, H. Ladurner & Peintner) Klofac	MK028949	99.70	UDB002181	Zajączek
WA0000071098	*Imleria badia* (Fr.) Fr.	a.f.	–	–	Podgrzybek
WA0000071099	*Imleria badia* (Fr.) Fr.	MK028950	100	KX756408	Podgrzybek
WA0000071100	*Xerocomellus pruinatus* (Fr. & Hök) Šutara	MK028951	100	UDB000008	Zajączek
WA0000071101	*Imleria badia* (Fr.) Fr.	a.f.	–	–	Podgrzybek

*a.f.* molecular analysis failed

From our interviews and field observations, we hypothesize that most of the taxa not recorded in the 1960s were overlooked rather than being new uses. The local inhabitants are very conservative and cautious about fungi use and field guides tend to be used to confirm the identification of already-collected species. They usually do not start collecting new species based on the field guide. Of course, some new uses cannot be excluded. One of the respondents learned to use puffballs while receiving visitors from the UK and applied the English name, “puffball” on an everyday basis! Another example is *Pleurotus ostreatus*, which has not been traditionally consumed in Poland and was not present in the guide written by Orłoś [37]. Its collection from the wild became popular in the last few decades because of its broad commercial use and its presence in many modern culinary recipes.

### Changes in preferences concerning wild fungi collection

**Fig. 7:**
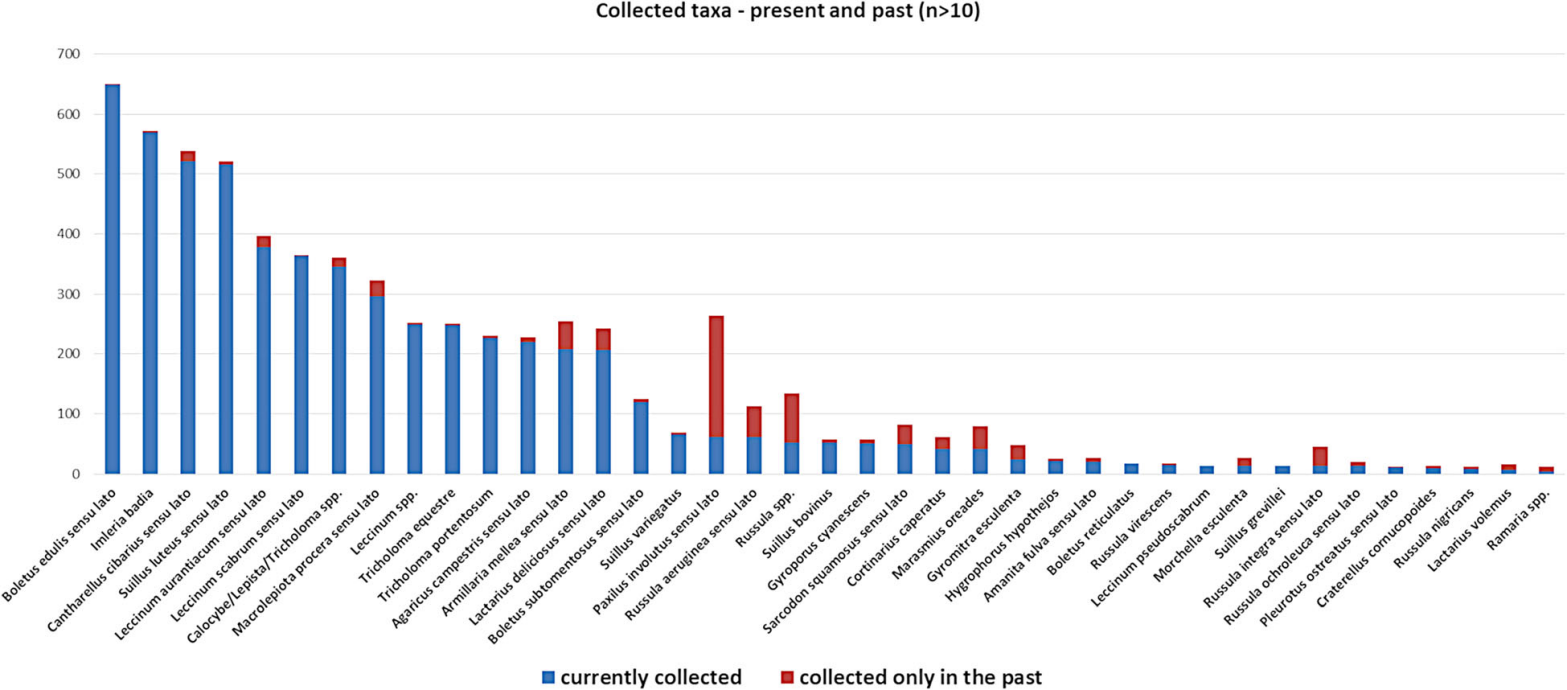
Fungi taxa collected currently (blue) and only in the past (red)

Among taxa listed as edible by Mazovian inhabitants, a few species are currently considered as poisonous in Poland. These are *Paxillus involutus*, *Amanita muscaria*, *Gyromitra esculenta*, and *Scleroderma citrinum* [38, 39]. It is worth noticing that *P. involutus* is regarded as an edible mushroom by 38% of respondents. The reason behind this is that *P. involutus* was traditionally used as food in Mazovia until the 1980s, when the first reports about *Paxillus* poisoning syndrome were published in Poland [15]. The data on which taxa are used as food currently and which were used only in the past enable the depiction of changes in preferences concerning wild fungi collection. By comparing this data, we can see that the majority of the respondents stopped collecting *P. involutus* after warnings about their toxicity. However, 9% of them still claim that *P. involutus* consumption is perfectly safe (Fig. 7).

*Gyromitra esculenta* and *Scleroderma citrinum* are usually consumed after specific preparation. *Gyromitra esculenta* is allowed for commercial use in Finland where it is considered a delicacy. However, it is sold only with attached instructions for its preparation [40]. Young and dried *Scleroderma* is used only as food flavoring, and according to some reports, it is safe to consume in very small portions [41]. It is usually used as a substitute for *Tuber* species; however, it is generally perceived as mildly toxic and unsafe for consumption [39, 42]. According to collected reports, *A. muscaria* was only used as food in the region in the past, during periods of war and famine, after long boiling and discarding of the water. This enabled the removal of toxins from its fruiting body. This method of preparation was also recorded in Italy [43, 44]. The memory of the use of *Amanita muscaria* was recorded in the villages of Klusek, Kozietuły, Leksyn, Psucin, and Stare Babice. While analyzing data concerning differences between taxa collected currently and in the past, we can also notice the large decline in the collection of species from *Russulaceae* family. This can be correlated with their absence on the list of fungi species allowed for commercial use in Poland [45], although they used to be widely collected (e.g., [14]).

### Cultural significance

According to Smith’s Salience Index, the most culturally significant edible fungi taxa are *Boletus edulis* sensu lato (0.9157), *Imleria badia* (0.7959), *Cantharellus cibarius* sensu lato (0.7387), *Suillus luteus* sensu lato (0.7020), and *Leccinum aurantiacum* sensu lato (0.5368). The most salient inedible or poisonous taxa are *Amanita* sp. (0.4804), *Tylopilus felleus* (0.3666), *Amanita muscaria* (0.3048), and *Amanita phalloides* sensu lato (0.2767). Fungi taxa with other than culinary uses are characterized by low salience values. The most salient among them are *Amanita muscaria* (0.0950), *Polyporales* sp. (0.0187), *Piptoporus betulinus* (0.0158), *Psilocybe* sp. (0.0084), and *Gyromitra esculenta* (0.0027).

### Folk taxonomy

The large number of interviews and frequent interaction with mushroom collectors enabled the transcription of local folk taxonomy into proper taxonomic classification. The acquired information enabled us not only to assign folk taxa to scientific taxonomic nomenclature, but also to describe folk views on connections between particular taxa.

The majority of folk fungi classifications in the study area taxa were based on units defined as folk genera [46] (or generic species [47]). Sometimes, these folk genera were universally divided into two or more folk species using folk binominals (e.g., in the case of *Leccinum*). Usually one, the most frequent, of the scientific species was taken as the model (“core”) of the folk genus representing its “essence” (compare [47]) and a few more closely related species from the same section were classified in the same folk genus. However, there were also instances when informants were able to distinguish other species with different local names from the core taxon based on model species. These species were divided in two groups—in a broad sense (sensu lato) and in a strict sense (sensu stricto). One such example is “prawdziwek” (porcini), identified as *Boletus edulis* sensu lato, within which some respondents were able to distinguish “prawdziwek dębowy” (oak porcini)—*Boletus reticulatus*, and “prawdziwek piaskowy” (sand porcini)—*Gyroporus castaneus*, though most respondents would not distinguish them. There were also cases when informants were able to distinguish a group consisting of separate taxa whose fruiting bodies had a similar appearance. This occurred with the taxa named “kozaki,” which corresponds to the *Leccinum* genus. Within this taxon, on the basis of different coloring, two model species, *Leccinum aurantiacum* sensu lato and *Leccinum scabrum* sensu lato were distinguished. Within the collective taxon *Leccinum aurantiacum* sensu lato, some of the respondents distinguished *L. quercinum*, *L. versipelle*, and *L. vulpinum*. Furthermore, within the group of *L. scabrum* sensu lato, 14 respondents were able to distinguish *L. pseudoscabrum*. All these species were differentiated on the basis of such characteristics as color, symbiotic relations, flesh characteristics (discoloration and density), and habitat. A similar model of classification applies to other genera such as *Russula*.

Classification of fungal species on the basis of the shape of fruiting bodies does not always coincide with one individual scientific genus. This happens in the case of folk taxa, known across most of the Mazovia region under the name “gąski” (literally “geese”). Because of the similarity in the shapes of their fruiting bodies, this folk taxon consists of three genera—*Calocybe*, *Lepista*, and *Tricholoma*. Within this taxon, Mazovian inhabitants identify species such as *Calocybe gambosa*, *Lepista nuda*, *Tricholoma equestre*, and *Tricholoma portentosum*. This was observed in the villages of Korytów, Klusek, Szczaki, and Węgrzynowice.

In the case of species from the genus *Suillus*, the majority of collected species are associated with the model species *Suillus luteus*. In folk taxonomy, *Suillus variegatus* is not perceived as a species associated with other *Suillus* species, and has different names, due to its distinctive form.

Among inedible and poisonous fungi (Table 2), a different group, which cannot be fully assigned to existing scientific taxa, is the mushrooms known as “psiaki” (literally “dog mushrooms”). This folk taxon contains all species with small fruiting bodies belonging to the *Agaricales* order. Another higher taxon distinguished in folk taxonomy is “huby,” (bracket fungi) which can be assigned to the order *Polyporales* (Tables 2 and 3). *Rubroboletus satanas* was described as poisonous by five respondents despite its absence in the local mycobiota. In this case, literature was the main source of their knowledge, as this species gained notoriety across the country as the most poisonous *Boletaceae* that can be found in Polish forests.

On the basis of collected data about the folk methods of fungi classification, we can determine the main factors responsible for folk fungi taxa differentiation. These are:

1. Order/family/genus—shape of fruiting bodies;
2. Species (in a broad sense)/section—shape, color, utilitarian properties; and
3. Species (in a strict sense)—shape, color, utilitarian properties, symbiotic relations, habitat, time of occurrence, taste, smell, flesh characteristics, milk presence, and characteristics.

### Differentiation of local fungi names

Data acquired during folk taxonomy analysis enabled us to collect 526 folk names of wild growing fungi. There is visible discrepancy in number of local names assigned to particular fungi taxa. For example, 397 respondents, who have traditional knowledge about *Leccinum aurantiacum* sensu lato collection, listed 25 different local names of this fungus while 242 respondents who listed *Lactarius deliciosus* sensu lato know this taxon only under one name—“rydz”.

### DNA barcoding

Edible fungi samples collected during field research were used to further DNA barcode analysis. Out of 101 samples, 88 were successfully identified using molecular analysis (Table 4). Sixty-four samples came from voucher specimens collected fresh during field research, and 24 were acquired from already dried specimens preserved by the respondents. As many as 11 of analyzed samples were not identified during previous field research; thus, the number of fungi taxa identified during present research increased to 92. Among species identified using DNA barcoding are two (*Hydnum elipsosporum* and *Paxillus cuprinus*) that are new to the mycobiota of Poland [48–50]. Identification of these species among other edible fungi collected by people living in the Mazovia region is also the first direct confirmation of their use for consumption.

### Comparison of the results with available data

The majority of regional ethnomycological studies have focused only on fungi species used for consumption. Examples include works from Mexico, such as the study conducted in two municipalities of the Sierra Tarahumara, with 22 recognized edible folk taxa [51]; in Tsotsil town in the Highland of Chiapas with 25 edible taxa [52]; or in Amelaco, Quéretaro, where the authors were able to list 33 taxa [53]. The number of species sold in local markets in Mexico is much higher. For example, in the Ozumba market, 60 different species of fungi are sold throughout the year [54]. The same number of species was reported as sold in the markets in the city of Poznań (Poland) in the 1930s [11]. Other recent works come from the western Black Sea region of Turkey (33 edible species) [7] and Africa, such as studies from Cameroon with 22 edible fungi taxa [55], or the research conducted by Tibuhwa in rural areas of Tanzania where 75 different wild fungi species were recorded as sold as food in local markets [6]. In the case of the present research, by using both species identification in the field and DNA barcode identification, we were able to compile a total list of 76 different fungi species used as food by people living in the Mazovia region. This is the longest list of edible fungi species recorded during field ethnomycological research (one species more than the list from Tanzania). Furthermore, the complete list of 92 fungi taxa (including inedible and poisonous and taxa with other than culinary purposes) listed both during field research and molecular identification is simultaneously the longest list recorded during ethnomycological studies based on field research.

Although mushroom collecting in Poland is common and culturally salient, in other areas of Poland, only shorter lists composed of 20–30 species are known [14, 56]. Obviously, the extent of our study was relatively large, facilitating the obtaining of a longer list, but cultural factors also may play a role. Unfortunately, we do not have detailed comparative data from other Northern Slavic countries. From our preliminary unpublished observations and popular literature on fungi use, we can hypothesize that all these countries (Czech Republic, Poland, Slovakia, Belarus, Ukraine, and Russia) form something which we call the “Northern Slavic Mycophilic Belt.”

### Characteristic species documented during the research

Among fungi recorded as used for consumption purposes in the Mazovia region are a few species whose collection is restricted only to particular locations. For example, *Scleroderma citrinum* is used as a spice for food only in villages situated in central and south-eastern Mazovia, e.g., Burakowskie, Całowanie, Łękawica, Stare Babice, and Żurawka. The collection of *Calvatia gigantea*, which was, by the way, protected in Poland until 2014, is popular in Flesze village, which is the northernmost studied location. The fruiting body of this fungus is usually cut into thick slices and pan-fried coated in grated breadcrumbs and egg. *Calocybe gambosa* is gathered in the south-western Mazovian villages of Korytów and Węgrzynowice. This species is prepared for consumption in the same way as species from the genus *Tricholoma*. It is used as an ingredient in soups and sauces and as a side-dish after pickling in vinegar. Residents of the south-western villages Korytów and Pszczonów often collect *Craterellus cornucopioides*, which is usually sautéed with scrambled eggs and used as a sauce ingredient. It is also considered a great filling for *pierogi* dumplings. We can also notice an interesting distribution of localities concerning the frequent collection of *Hygrophorus hypothejus*. The use of this species is very popular in two villages situated near the south-western border of the Mazovia region (Pszczonów, Węgrzynowice) and two villages located in the north-eastern part of Mazovia (Cieciory, Wyrzyki). This species is usually consumed as a snack after pickling in vinegar, but it can also be used as an ingredient in everyday dishes. The village of Węgrzynowice is the only location with a record of *Lactarius piperatus* consumption, which was used as a food only after boiling and discarding the water. *Lactarius vellereus* is most popular in the village of Psucin where its fruiting bodies, after a long soaking in water, are salt-fermented in a large metal vessel (called *sagan*). Futhermore, the village of Dąbrowa is the only one in which inhabitants distinguish *Leccinum quercinum* species from other orange-capped *Leccinum* species, and it is considered as a delicacy on a par with *Boletus edulis*.

It is worth mentioning that men are significantly more knowledgeable about wild edible fungi species than women (Fig. 3). This opposes the general view on wild fungi pickers based on 80 ethnomycological studies with gendered data [57]. A similarly greater mushroom knowledge among men was previously recorded in Poland [56] and was also observed in China [58].

## Conclusion

Evenly dispersed research localities and a large number of individual interviews enabled the documentation of an as yet unrecorded scope of local knowledge of 92 wild fungi taxa. This is the longest list of wild fungi ever recorded during ethnomycological research. The list includes 76 species used for consumption purposes, which is also the longest list of taxa used as food in any region on Earth. Among the taxa considered edible or conditionally edible, we can find species that are currently considered poisonous in Poland (*Amanita muscaria*, *Gyromitra esculenta*, *Paxillus involutus*, and *Scleroderma citrinum*) [38], partly protected (*Morchella conica* s.l., *Morchella esculenta*) [59], rare (*Boletus ferrugineus*, *Gyroporus castaneus*, *Gyroporus cyanescens*, *Chlorophullum olivierii*, *Leccinum variicolor*, *Leccinum vulpinum*, *Russula alutacea*, *Sparassis crispa*, *Xerocomellus cisalpinus*, *Xerocomellus pruinatus*) [60, 61], and even absent in available checklists of macrofungi found in Poland (*Hydnum ellipsosporum*, *Paxillus cuprinus*) [48, 61]. These results confirm the highly mycophillic character of Mazovian food culture and encourage research in adjacent areas of Poland, Belarus, and Ukraine.
